# Childhood sigmoid volvulus

**DOI:** 10.12669/pjms.42.9.19557

**Published:** 2026-09

**Authors:** Mesud Fakirullahoglu, Metin Yildiz, Rifat Peksoz, Esra Disci, Sabri Selcuk Atamanalp

**Affiliations:** 1 Mesud Fakirullahoglu, MD. Assistant Professor, Department of General Surgery, Erzurum City Hospital, Erzurum, Türkiye; 2 Metin Yildiz, MD. Assistant Professor, Department of General Surgery, Faculty of Medicine, Ataturk University, Erzurum, Türkiye; 3 Rifat Peksoz, MD. Associate Professor, Department of General Surgery, Faculty of Medicine, Ataturk University, Erzurum, Türkiye; 4 Esra Disci, MD. Associate Professor, Department of General Surgery, Faculty of Medicine, Ataturk University, Erzurum, Türkiye; 5 Prof. Sabri Selcuk Atamanalp, Department of General Surgery, Faculty of Medicine, Ataturk University, Erzurum, Türkiye

**Keywords:** Sigmoid colon, Sigmoid volvulus, Childhood

## Abstract

**Objectives:**

Childhood sigmoid volvulus (SV) is a comparatively rare illness. The objective of this study was to examine the recent advances in childhood SV.

**Methodology:**

In clinical review conducted at Ataturk University, Turkey, we used combined retrospective (from June 1966 to July 1986) and prospective (from July 1986 to July 2026) evaluation.

**Results:**

Fourteen children constituted 1.3% of total 1,101 SV cases. Mean age was 10.3 years (range: 10 weeks-17 years) and male/female ratio was 12/2 (85.7%/14.3%). Previous SV attack was present in two patients (14.3%). Mean admission period was 60.9 hours (range: 24-96 hours). Main clinical features were abdominal pain/tenderness (12 cases, 85.7%), distention (ten cases, 71.4%), abnormal bowel sound (nine cases, 64.3%), vomiting (seven cases, 50.0%), obstipation and muscular rigidity/rebound tenderness (six cases, 42.9% for each), hypokinetic/akinetic bowel sound and shock (four cases, 28.6% for each), and gangrenous stool (two cases, 14.3%). SV was diagnosed by abdominal X-ray radiography in seven patients (50.0%), at laparotomy in five cases (35.7%), and by computed tomography in two patients (14.3%). Emergency surgery was required in 12 cases (85.7%), while endoscopic detorsion was recently performed in two cases (14.3%) and elective surgery was added in one (7.1%). Mortality and morbidity were seen in four cases (28.6%) for each.

**Conclusion:**

Although childhood SV is rare, it is still a critical area due to its relatively poor anamnestic inability, diagnostic difficulty, and prognostic outcome. However, diagnostic efficacy of CT, therapeutic contribution of endoscopic detorsion, and recurrence-preventive effect of elective laparoscopic surgery are important advances during the recent years.

## INTRODUCTION

Sigmoid volvulus (SV) is a comparatively rare disease in childhood.^1^ Although this clinical entity is not a mystery, limited number of papers makes it a highly controversial topic.^2^,^3^ Complex clinical course, inadequate medical history, and underachieving physical examination findings appear as current problems.^4^,^5^ However, advanced diagnostic tools, current treatment options, and improved pre- and postoperative care opportunities are crucial developments in this area.^6^,^7^

Contrary to relatively rare frequency of SV in western countries, which incidence is 1.67 patients per 100,000 persons per year in the United States, it is not an unknown disease for the practitioners in our region, Eastern Anatolia, with an incidence of 4.1 per 100,000 person-year.^8^,^9^ In this article, in the strength of the largest mono-center SV series including total 1,101 cases treated over 59.5 years (from June 1966 to January 2026), we aimed to investigate the clinical characteristics of 14 children with SV and to discuss the recent advances in childhood SV.^8^

## METHODOLOGY

In clinical review, we used combined retrospective (total 612 patients, 55.6%, from June 1966 to July 1986) and prospective (total 489 patients, 44.4%, from July 1986 to January 2026) evaluation. Among total 1,101 SV patients, childhood (patients younger than 18 years) SV cases were included this study. We noted age, gender, previous SV history, admission period, symptoms and signs, treatment options, mortality, and morbidity in each case.

In the diagnosis of SV, together with clinical consideration, we used abdominal X-ray radiography and computed tomography (CT) in recent years. Although emergency surgery was the sole option in all cases in early years, we preferred nonoperative detorsion (flexible endoscopy) as the first line treatment in uncomplicated cases in recent years, while emergency surgery (detorsion, mesopexy, or sigmoidectomy with stoma or primary anastomosis) was required in the presence of bowel gangrene or unsuccessful endoscopic detorsion.We used numbers and percentages in analysis and evaluation.

### Ethical Approval:

Institutional review board (Ethical Committee of Ataturk University Faculty of Medicine, 03/25, dated: January 31, 2025) and informed consents were present for this study.

## RESULTS

As demonstrated in Table-I, when cases with ileosigmoid knotting are excluded, 14 children (1.3% of total 1,101 cases) with 10.3 years of mean age (range: 10 weeks-17 years) and 12/2 (85.7%/14.3%) of male/female ratio were treated. A history of previous SV treated with endoscopic detorsion was present in two patients (14.3%). Mean admission period was 60.9 hours (range: 24-96 hours). Main clinical features were abdominal pain/tenderness in 12 patients (85.7%), distention in ten (71.4%), vomiting in seven (50.0%), obstipation and muscular rigidity/rebound tenderness in six (42.9%) for each, hyperkinetic bowel sound in five (35.7%), hypokinetic/akinetic bowel sound and shock in four (28.6%) for each, and gangrenous stool in two. Diagnostic procedure was abdominal X-ray radiography in seven cases (50.0%), CT in two, and laparotomy in five.

**Table-I:** Data on 14 children with sigmoid volvulus.

Parameter	Result	
Age (mean year, range)	10.3 (10 weeks-17)	
Gender (male/female)	12/2 (85.7%/14.3%)	
Previous SV history	2 (14.3%)	
Admission period (mean hour, range)	60.9 (24-96)	
Clinical features	Abdominal pain/tenderness	12 (85.7%)
	Distention	10 (71.4%)
	Vomiting	7 (50.0%)
	Obstipation	6 (42.9%)
	Muscular rigidity/rebound tenderness	6 (42.9%)
	Hyperkinetic bowel sound	5 (35.7%)
	Hypokinetic/akinetic bowel sound	4 (28.6%)
	Shock	4 (28.6%)
	Gangrenous stool	2 (14.3%)
Diagnostic procedure	X-ray radiography	7 (50.0%)
	Computed tomography	2 (14.3%)
	Laparotomy	5 (35.7%)
Therapeutic procedure	Endoscopic detorsion	2 (14.3%)
	Alone	1 (7.1%)
	Plus elective sigmoidectomy and anastomosis	1 (7.1%)
	Emergency surgery	12 (85.7%)
	Sigmoidectomy and stoma	8 (57.1%)
	Surgical detorsion	2 (14.3%)
	Sigmoidectomy and anastomosis	1 (7.1%)
	Surgical detorsion and sigmoidopexy	1 (7.1%)
Mortality		4 (28.6%)
Morbidity		4 (28.6%)

Successful endoscopic detorsion was performed in two cases are demonstrated in Fig.1. Following endoscopic detorsion, elective laparoscopic sigmoidectomy and primary anastomosis was applied in one patient (7.1%). Emergency surgery was required in 12 cases. In nine patients (64.3%) with gangrenous bowel, stoma or primary anastomosis was used in eight cases (57.1%) and one case, respectively. In three cases with viable bowel (21.4%), surgical detorsion alone or additionally sigmoidopexy was performed in two patients and one patient, respectively.

**Fig.1:**
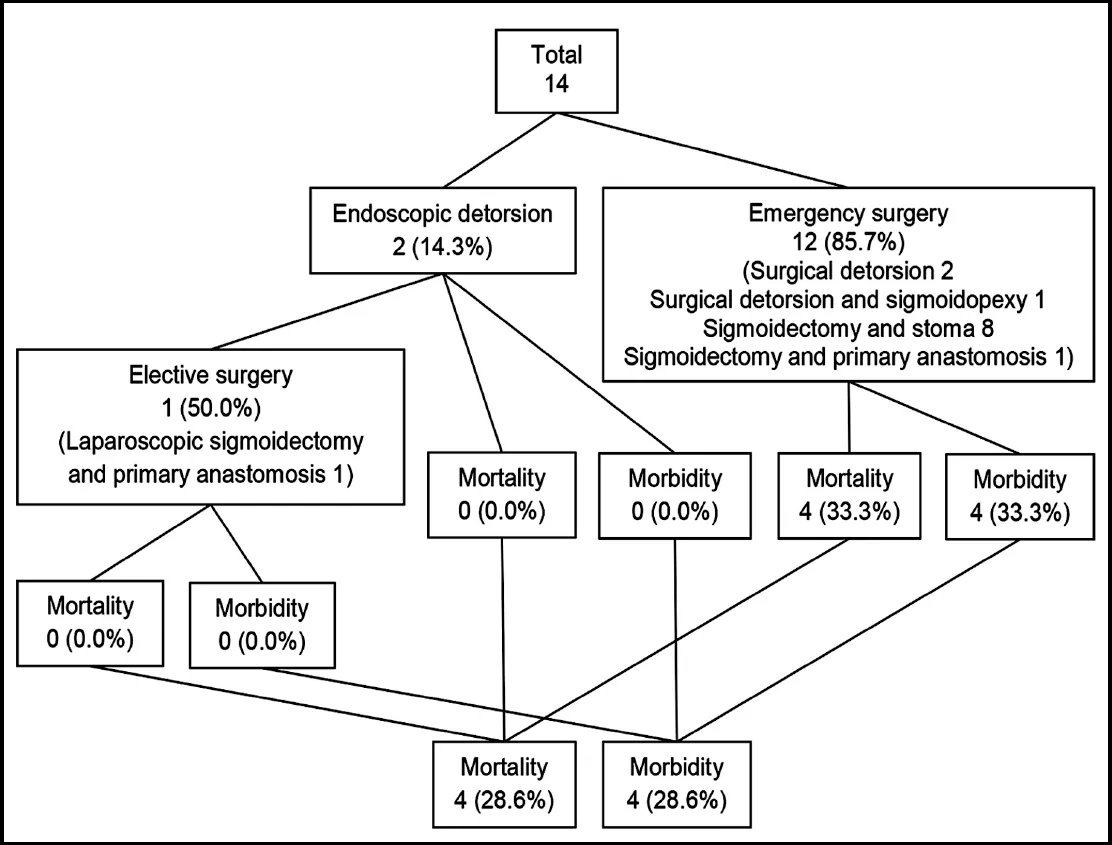
Treatment options and prognosis in 14 children with sigmoid volvulus.

Mortality was determined in four cases, all of which were in emergency surgery group. Additionally, all of mortal cases had toxic shock, while two patients had bowel gangrene. Morbidity (wound infection in three, anastomotic leakage in one) were determined in four patients.

## DISCUSSION

Among less than one thousand cases reported to date in the worldwide literature, in 1990, Smith et al.^10^ presented a total of 48 child patients with SV (mean age: eight years) reported in the worldwide literature by that time. The following largest series from all over the world were reported by Salas et al.^11^ in 2000, which was consisted of 63 patients (mean age: seven years, range: four hours-18 years) and by Hencke and Loff^12^ in 2023, in which 256 cases (mean age: 10.2 years, range: one day-18 years) were evaluated. In 2024, Durkac et al.^4^ published 223-case childhood and young adult SV series (mean: 16 years, range: one month-20 years) obtained from multicenter data in the United States.

In our series, 14 childhood SV patients constituted 1.3% of the total 1,101 SV cases, while the youngest patient was a ten-week-old male. The male/female ratio was 6/1. Although we treated two (0.2%) and five (0.5%) young adults or adults with Hirschsprung’s disease and mental retardation, respectively, there were no cases with the aforementioned comorbidities in the childhood SV series. Our findings related to clinical presentation, diagnosis, treatment, and prognosis were compatible with the findings in the literature.

In ISK, a four-hour baby was reported to be the youngest patient in the world literature.^11^ Most likely due to anatomical inclination, similar to that of the adults, SV is frequently encountered in men children in childhood.^4^,^10^-^13^ Male/female ratio was reported to be 1.2/1-3.5/1 in childhood SV.^4^,^11^,^12^

An important characteristic of childhood SV is the association of SV with different physiological conditions and congenital or acquired diseases. Although rare in total, pregnancy is the most common physiological comorbidity in adult SV.^14^ Regarding the pathological entities in childhood, Hirschsprung disease was reported to be present in 10%-15.9% of the cases.^11^,^12^,^15^,^16^ Similarly, the incidence of neuromuscular diseases was reported to be 7.2% by Durkac et al.^4^ Other relatively uncommon concomitant diseases are mental retardation and Chagas’ disease.^16^-^18^

In childhood SV, clinical course is complex and diagnosis generally depends on clinical suspicion.^1^,^5^ In this age group, late admission, which was reported to be mean 1.5 days (range: two hours-four days), is an important problem.^5^,^11^ Late admission and diagnosis generally ensues from the complexity of the disease, inadequate anamnesis due to problematic contact, and insufficient physical examination findings.^5^ Chronic constipation may be the only history in the past of most patients.^6^ The rate of chronic constipation anamnesis was reported to be 10.2% by Hencke and Loff,^12^ 33.8% by Durkac et al,^4^ and 46.2% by Colinet et al.^17^ Similar to that of the adults, other most common features are abdominal pain/tenderness (41%-100%) and distention (56%-85%) in childhood SV.^10^-^12^,^17^,^19^ Short-term obstipation is another common symptom, which was reported to be in 10%-33.8% of the cases.^1^,^4^,^10^,^17^ In some children, contrary to the expectations, vomiting and nausea may be the subsequent symptoms with rates of 31%-59% and 5%-53.8%, respectively, and obstipation may be encountered in a small part of the patients.^10^-^12^,^17^ When compared with that of the adults, diarrhea is another common symptom in children, which was presented in 8%-23% of the cases.^11^,^17^ In our experiment, a careful anamnesis may present a few little-amount diarrhea as a prodromal symptom before the obstruction, and probably as a triggering factor of volvulus. A less frequent sign is abdominal mass in 5%-10% of the cases.^10^,^11^ As an indicator of bowel ischemia, signs of peritonitis, was reported to be present in 23.1%-59% of children with SV.^12^,^17^

Although abdominal X-ray radiographs reveal a coffee bean sign in approximately 29%-65% of the cases, the success rate is generally under 30% in childhood series.^5^,^10^-^12^,^17^ For this reason, computed tomography (CT) is the most commonly preferred diagnostic tool with mesenteric whirl sign in addition to X-ray findings with 56%-98% of diagnostic accuracy rates in recent years.^1^,^12^,^17^-^19^

A rapid and effective resuscitation is essential in the management of childhood SV.^1^,^5^,^12^,^20^,^21^ Complicated patients require emergency surgery including sigmoid colectomy and primary anastomosis or stoma in gangrenous cases, while detorsion alone in patients with viable bowel.^5^,^6^,^11^,^12^,^20^,^21^ Although conventional treatment option was emergency surgery in all cases in the past years, based on commonly usage of the pediatric endoscopes, recent publications present endoscopic detorsion as the first line option in cases with accurate diagnosis and without bowel gangrene or peritonitis with 47%-100% of success and 0%-1% of morbidity rates.^6^,^12^,^17^,^22^,^23^ Barium enema has a historical significance due to perforation and missing of the bowel ischemia.^5^,^11^ However, due to the presence of a redundant sigmoid colon with a long and a narrow-based mesentery in almost all cases, SV tends to recur in 38%-57% of all cases.^1^,^12^ Moreover, when compared with that of the adults, children SV cases are under a higher recurrence risk arising from early onset, in whom recurrence rate is reported to be 46.2%-100%.^12^,^13^,^17^,^22^ For this reason, elective sigmoid colectomy followed by endoscopic detorsion, which prevents the recurrence risk, may be the optimal treatment plan in almost all cases except for those with a high surgical risk.^6^,^7^,^10^-^12^,^17^ On the other hand, mezosigmoidopexy and sigmoidopexy, which reduce the recurrence risk, may be other alternatives.^7^,^12^,^23^,^24^ It is well known that, laparoscopic procedures have some undisputed advantages in comparison to open surgery when performing the surgical procedures.^21^,^23^,^25^

In childhood SV, a high risk of complications such as bowel ischemia, necrosis, perforation, or peritonitis arising from missed or delayed diagnosis, worsens the prognosis.^1^,^11^,^12^ Although the overall mortality was over 50% in the past years, it has recently decreased to 10%.^1^,^12^ Mortality rate was reported to be 3.1% by Durkac et al,^4^ 3.5% by Hencke and Loff,^12^ and 25% by Smith et al.^10^ Morbidity rate is 4%-30%.^5^ In childhood SV, median hospital stay was reported to be seven days (range: 5-12 days) by Durkac et al.^4^

### Limitations

Limited availability of childhood SV series, partial retrospective evaluation, and heterogeneity due to very long study period are salient limitation points of this study. However, large-series prospective studies require any number of childhood SV cases, multicenter information sharing, and several years.

## CONCLUSION

Although relative diagnostic difficulty arising from poor anamnestic ability is still a problem in the diagnosis of SV in childhood, which is a cause of poor prognosis, the use of CT may recently facilitate the correct diagnosis. Additionally, the utility of pediatric endoscopes may provide an opportunity for both diagnosis and treatment in patients without bowel ischemia or perforation, and elective laparoscopic sigmoid colectomy may reduce SV recurrence in selected cases. However, further large-scale or multicenter prospective studies are required to interpret this rare clinical entity.

### Authors’ Contribution:

**MF & SSA:** Data collection, manuscript writing.

**MY, RP and ED:** Data collection, revision of the final draft.

**SSA** is responsible and accountable for the accuracy or integrity of the work.
